# Does service timing matter for psychological outcomes in cardiac rehabilitation? Insights from the National Audit of Cardiac Rehabilitation

**DOI:** 10.1177/2047487317740951

**Published:** 2017-11-09

**Authors:** Jennifer Sumner, Jan R Böhnke, Patrick Doherty

**Affiliations:** 1 Department of Health Sciences, University of York, UK; 2 Saw Swee Hock School of Public Health, National University of Singapore, Singapore; 3 School of Nursing and Health Sciences (SNHS), University of Dundee, UK

**Keywords:** Anxiety, depression, cardiac rehabilitation, audit

## Abstract

**Background:**

The presence of mental health conditions in cardiac rehabilitation (CR) patients such as anxiety and depression can lead to reduced programme adherence, increased mortality and increased re-occurrence of cardiovascular events undermining the aims and benefit of CR. Earlier research has identified a relationship between delayed commencement of CR and poorer physical activity outcomes. This study wished to explore whether a similar relationship between CR wait time and mental health outcomes can be found and to what degree participation in CR varies by mental health status.

**Methods:**

Data from the UK National Audit of Cardiac Rehabilitation, a dataset that captures information on routine CR practice and patient outcomes, was extracted between 2012 and 2016. Logistic and multinomial regression models were used to explore the relationship between timing of CR and mental health outcomes measured on the hospital anxiety and depression scale.

**Results:**

The results of this study showed participation in CR varied by mental health status, particularly in relation to completion of CR, with a higher proportion of non-completers with symptoms of anxiety (5% higher) and symptoms of depression (8% higher). Regression analyses also revealed that delays to CR commencement significantly impact mental health outcomes post-CR.

**Conclusion:**

In these analyses CR wait time has been shown to predict the outcome of anxiety and depression status to the extent that delays in starting CR are detrimental. Programmes falling outside the 4-week window for commencement of CR following referral must strive to reduce wait times to avoid negative impacts to patient outcome.

## Introduction

An estimated 85 million people in Europe live with cardiovascular disease.^1^ As survival rates improve, following acute cardiac events, this number is only set to rise.^2^ Although improvements in life expectancy are positive, with increasing age multimorbidity i.e. living with more than one chronic condition becomes more common.^3^ For example frequently those with chronic conditions experience mental health problems such as depression and anxiety.^4^ A systematic review of depression prevalence in acute myocardial infarction survivors reported major depression was present in 19.8% of the population and the proportion with significant symptoms varied between 15% and 31% depending on the type of screening instrument used.^5^ Comorbid depression and anxiety are especially concerning: impacting quality of life, persisting for long periods of time, are associated with increased healthcare costs^6,7^ and elevated mortality.^4,8,9^ A higher lifetime risk of depressive or anxiety disorders has also been observed in those with a history of cardiovascular disease.^10^

In light of increasingly multimorbid populations cardiac rehabilitation (CR) has long since shifted from its origins as a pure exercise regime. In 2000 the national service framework for coronary heart disease was published in the UK, detailing modern standards of care, including CR services.^11^ This was followed in 2003 by a position statement by the European Society of Cardiology, which provided recommendations on the design and development of CR programmes.^12^ CR in Europe is now expected to be multi-component and multidisciplinary typically including education and psychological support.^13^ As part of modern practice, baseline assessments including the hospital anxiety and depression scale (HADS)^14,15^ are conducted upon enrolment to CR in the UK. The HADS has been shown to be appropriate for screening and as a patient-reported outcome in cardiac populations.^16^ Its use means participants’ care can be tailored to the needs of the individual patient such as providing psychological support.

For successful CR appropriate management of mental health conditions is critical.^17^ The presence of anxiety or depression may exacerbate the underlying cardiac condition through reduced programme adherence, lower use of medical care and the pursuit of unhealthy behaviours such as smoking.^4,18^ The presence of anxiety and depression has also been linked to increased mortality and re-occurrence of cardiovascular events.^19–22^ Thus, ineffective identification and treatment of comorbid depression and anxiety undermines the goals of CR.^23^

In order to deliver successful CR it is important to identify factors which impact mental health. Previous research on CR services has found associations between CR wait time and physical activity outcomes, showing that longer wait times significantly reduce the likelihood of improvement in fitness-related measures.^24^ In this study we explore whether programme delivery, in particular timing, may also impact mental health outcome and how participation in CR may differ between symptomatic and non-symptomatic patients. In particular, this study investigates the participation of patients eligible for CR with and without symptoms of anxiety and depression and whether delays in initiating care predict mental health outcome following CR, measured using HADS.

## Methods

This study is reported according to the strengthening the reporting of observational studies in epidemiology (STROBE) checklist.^25^ In the UK CR is delivered in accordance with national standards and for most patients includes centre-based CR (80%) with an emerging trend for home-based self-management approaches.^13,26–28^ Ideally, programmes should run for 12 weeks twice weekly and consist of multiple components: physical activity, education, dietary modification and psychological support.^13,29,30^ Data on service delivery, utilisation, patient characteristics and their respective outcomes are entered onto the National Audit of Cardiac Rehabilitation (NACR) by practitioners involved in CR delivery, according to a data dictionary (http://www.cardiacrehabilitation.org.uk/nacr/downloads.htm). Participation in NACR is high: in 2016 an average of 72% of all CR programmes entered data onto the NACR dataset.^31^ Typically, CR-indicated patients are approached by the CR team and referred to the service while the patient is still in hospital after the acute treatment phase or shortly after discharge. For those that enroll a pre-assessment takes place, during which patient sociodemographic and clinical characteristics are recorded. Following completion of the CR programme the clinical assessment is repeated.

### Participants

Data from the NACR was extracted from 1 January 2012 to 31 August 2016. Adult patients (≥18 years) with acute coronary syndrome were included. During the study period 137,178 patients started core CR and 93,870 completed core CR. Patients who started CR and had a completed baseline HADS assessment were included in the investigation of CR participation (*N* = 56,233). A total of 39,588 patients started and completed CR and had both a baseline and post-CR HADS assessment. These patients formed the main analysis sample. For analyses of the association between CR wait time and mental health outcome missing data were imputed for those who started and completed CR in centres with data for least 10 patients, generating a sample of 92,086 for a sensitivity analysis.

### Measures

Current guidance states that patients should be seen early by the outpatient CR team and start CR within 4 weeks of referral, and ideally run for 12 weeks twice weekly.^13,26,29,32,33^ Three variables were defined to capture participation in CR: (a) wait time, i.e. time between referral to CR and start of CR; (b) duration of CR (days), i.e. between start and end date of CR exceeding 7 days; and (c) non-completion of CR defined as those with a CR start date entry but no completion date. For the regression analyses CR wait time (i.e. time between referral and CR start) was included as a continuous variable (days) to determine the impact on HADS outcome for each single day increase in CR wait time, and separately as a categorical variable to assess the impact of CR wait time according to current recommendations (on time 0–28 days, delayed 29–365 days). Some CR patients undergo more invasive surgical procedures as part of treatment such as bypass surgery, i.e. coronary artery bypass graft (CABG). For these patients timing categories were adjusted as recovery from surgery (e.g. sternotomy) takes longer and is an important step before rehabilitation can commence. For CABG patients timing groups were defined as ‘on time’ 0–42 days and ‘delayed’ 43–365 days.

The HADS^14^ is a screening tool for symptoms of anxiety and depression. It is typically self-completed by patients under the guidance of a trained medical professional. The HADS consists of 14 statements of which seven describe symptoms connected to depression (e.g. ‘I feel as if I am slowed down’) and seven are anxiety related (e.g. ‘I feel tense or wound up’). Patients respond on four categorical anchors (coded from 0 to 3). No individual item data were available to evaluate the reliability of HADS scores in the audit sample, but it has previously been found to be acceptable.^34^ The correlation between baseline and post-CR assessments was 0.73 (95% confidence interval (CI) 0.72–0.73).

In our main analysis anxiety and depression scores were analysed categorically (no symptoms/symptoms present) according to established clinical cut-offs with scores less than 8 representing low or no symptoms of anxiety or depression.^14,15^ Changes in HADS category between pre and post-CR were also derived and categorised as: (a) ‘symptomatic to non-symptomatic’; (b) ‘no change in symptomatic patients’; (c) ‘non-symptomatic to symptomatic’; (d) ‘no change in non-symptomatic patients’.

### Statistics

All analyses were conducted using STATA version 14.2. Summary statistics are presented as mean with standard deviation (SD), medians with interquartile ranges or percentages as appropriate. The median time until start of CR and duration of CR were calculated overall and by anxiety and depression classifications. Chi-squared or rank sum tests were used to investigate the statistical difference between symptomatic and non-symptomatic participants and a *t*-test was used to compare pre and post-CR HADS scores. Logistic regression analyses were performed to investigate the relationship between CR wait time and post-CR outcome (HADS category), and multinomial logistic regression models with ‘non-symptomatic to symptomatic’ as a reference category were used for change in anxiety and depression between pre and post-CR. Both analyses were adjusted for age, gender, number of comorbidities (0–5+) calculated from 19 prespecified comorbidity options as detailed in the NACR data dictionary (http://www. cardiacrehabilitation.org.uk/nacr/downloads.htm), CR duration, ethnicity (white British/other), relationship status (partnered/single), employment status (unemployed/employed/retired), history of previous cardiac event (present/absent), treatment received (revascularised/non-revascularisation), year of initiating event and baseline anxiety and depression score (for the CR wait time and post-CR outcome analyses only). As the data were clustered within CR centres we used cluster-robust standard errors to evaluate the significance of predictors. For the logistic and multinomial regressions missing data were also imputed via multiple imputation chained questions.^35^ The following variables were included in the imputation: age, gender, ethnicity, number of comorbidities, employment status, relationship status, CR duration, history of previous cardiac event, treatment received, year of event, and baseline and post-CR HADS scores. Twenty iterations were run and the quantity and pattern of missing data was assessed prior to imputation (detail presented in Table 1). To explore the relationship between wait time and HADS, marginal probabilities were calculated and explored visually. The amount of variance due to data clustering by centre was also explored using intraclass correlations for HADS scores, wait time and CR duration. Post-estimation checks were performed to investigate how well the statistical models fit to the data. Pearson chi-squared goodness-of-fit tests were performed to test whether there is a statistical difference between observed and expected values (for multinomial logistic regressions this was done using logistic regressions for all comparisons). In addition, for the logistic model specification tests were run^36^ to test whether non-modelled non-linear relationships were present.

**Table 1.** Patient characteristics.

	*N* = 39,588^a^	
Mean age, years (SD)	65.1 (SD 10.60)	
Gender, *n* men (%) *n* = 38,862	30,121 (78%)	
Ethnicity, *n* British (%) *n* = 33,149	28,697 (87%)	
One or more comorbidities, *n* (%)	29,326 (74%)	
Employment status, *n* (%) *n* = 33,894		
Employed	10,083 (30%)	
Unemployed	5184 (15%)	
Retired	18,627 (55%)	
Marital status: partnered, *n* (%) *n* = 30,823	24,769 (80%)	
Previous cardiac event, *n* (%)	13,108 (33%)	
Undergone previous revascularisation, *n* (%)	34,410 (87%)	
Median wait time to start CR from referral (days)	36 days (IQR 22, 57)	
Mean wait time to start CR from referral (days)	45 days (SD 38.26)	
Median CR programme duration (days)	59 days (IQR 47, 81)	
Mean CR programme duration (days)	67 days (SD 35.78)	
	Baseline	Post-CR
Symptoms of anxiety present, *n* (%)	11,015 (28%)	8394 (21%)*
Mean anxiety score (SD)	5.43 (4.04)	4.69 (3.77)*
Symptoms of depression present, *n* (%)	6734 (17%)	4637 (12%)*
Mean depression score (SD)	4.20 (3.50)	3.36 (3.22)*

SD: standard deviation; IQR: interquartile range; CR: cardiac rehabilitation.

a *N* = 39,588 unless otherwise stated.

*N* = 25,045 had data on all these variables.

* χ^2^ and *t*-test all *P* < 0.001.

### Ethics

The NACR is hosted by NHS Digital, through which designated researchers are approved to access anonymised patient-level data related to CR delivery processes and patient outcome pre and post-rehabilitation. These agreements are assessed annually as part of data governance approval between the NACR and NHS Digital. The aforementioned agreements and anonymity of the dataset meant that a separate ethical application was not required as part of this study.

## Results

### Cohort characteristics

Patient characteristics are presented in Table 1. A total of 39,588 patients completed CR and had a pre and post-CR HADS assessment. Participants were primarily men, were British, with a mean age of 65 years. The majority had at least one comorbidity, were in a relationship, were retired, had undergone previous revascularisation surgery and a third of participants had experienced a previous cardiac event. At baseline, 28% of patients had some symptoms of anxiety and a further 17% had symptoms of depression. Between the pre and post-CR period the proportion of symptomatic patients significantly decreased as well as the mean HADS scores.

In terms of data completion of the 56,233 patients who started and completed CR and had a completed baseline HADS assessment, 70% (*n* = 39,588) had a post CR HADS assessment entered onto the NACR dataset. Demographic characteristics between those who had a missing post-CR HADS assessment (*N* = 16,557) and those with a completed baseline and post-CR HADS assessment were similar; mean age 65.1 versus 64.2 years and the proportions for remaining demographics did not differ by more than 5% (data not shown).

We assessed the size of the clustering effect due to centres on our core variables in this analysis by determining intraclass correlations (ICC), which describe the amount of variance in these variables due to differences between the rehabilitation centres. The ICC for HADS depression scores at baseline was 0.02 (95% CI 0.01–0.02) and post-CR was 0.02 (95% CI 0.01–0.02), and the ICCs for HADS anxiety were 0.01 (95% CI 0.01–0.02) and 0.01 (95% CI 0.01–0.02) baseline and post-CR, respectively. The ICCs for wait time to start CR from referral (days) and CR programme duration (days) were 0.14 (95% CI 0.10–0.17) and 0.23 (95% CI 0.18–0.28), respectively. ICCs were small for HADS, indicating similar symptom distributions across rehabilitation centres, but ICCs were high for wait time and duration, which indicates by centre variation for wait time and duration. Since it has long been established that even small cluster effects can have detrimental impacts on statistical models,^37^ we proceeded with our strategy to use cluster-robust standard errors.

### Participation in CR

The median wait time for starting CR ranged between 36 and 37 days in those with or without symptoms of anxiety or depression. The duration of CR was 1 day longer in those with symptoms of anxiety (58 days) versus those without, and 4 days longer in those with symptoms of depression (61 days) versus those without (*P* < 0.001). The median wait time and CR duration are presented by a change in HADS category from pre to post-CR in Table 2. Wait time varied by no more than 2 and 4 days for change in HADS category for anxiety and depression, respectively. Duration of CR varied by 3 and 5 days for change in HADS anxiety and depression category, respectively. The proportion of non-completers was higher in those with symptoms of anxiety 28% versus 23% and higher in those with symptoms of depression 31% versus 23% in non-symptomatic patients (both *P* < 0.001).

**Table 2.** Median wait time and duration of CR by change in HADS anxiety and depression category.

Change in anxiety and depression category from baseline to post-CR	Change in anxiety category			Change in depression category		
	*N* (%)	Median wait time (days)	Duration of CR (days)	*N* (%)	Median wait time (days)	Duration of CR (days)
Symptomatic to non-symptomatic	4,880 (12%)	35	61	3,694 (9%)	36	63
No change in symptomatic patients	6,135 (16%)	36	60	3,040 (8%)	40	61
Non-symptomatic to symptomatic	2,259 (6%)	36	63	1,597 (4%)	37	63
Remains non-symptomatic patient	26,314 (66%)	37	58	31,257 (79%)	36	58

CR: cardiac rehabilitation; HADS: hospital anxiety and depression scale.

### CR wait time and outcome

**Figure 1.**
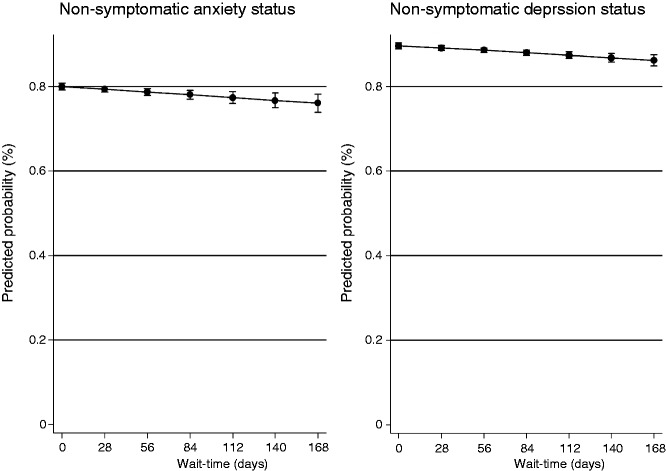
Predicted probability (%) of being non-symptomatic for anxiety and depression by wait-time (days).

Tables 3 and 4 present the results of the logistic and multinomial regression analyses. Statistically significant associations between HADS category (post-CR) and CR wait time were observed, i.e. increasing CR wait time increases the likelihood of symptomatic HADS anxiety or depression scores (≥8) post-CR. At a wait time of 28 days, the longest period starting CR would still be seen as on time, the predicted probability of being non-symptomatic for anxiety and depression was 79% and 89% decreasing to 76% and 86% by 168 days (6 months from referral), respectively (Figure 1). Testing model fit, Pearson chi-squared goodness-of-fit tests were non-significant (*P* = 0.92 and *P* = 0.90, respectively) and the specification tests revealed if at all only minor specification error.

**Table 3.** Results from logistic regression: CR wait time (late CR or CR wait time in days) and likelihood of being symptomatic following CR.

	Observed data		Imputed data	
	Anxiety symptoms OR (95% CI)	Depressive symptoms OR (95% CI)	Anxiety symptoms OR (95% CI)	Depressive symptoms OR (95% CI)
Late CR	OR 1.13 *P* = 0.002 (1.04, 1.23)	OR 1.24 *P* < 0.001 (1.12, 1.38)	OR 1.04 *P* = 0.07 (0.99, 1.10)	OR 1.09 *P* = 0.01 (1.01, 1.001)
CR wait time	OR 1.001 *P* = 0.001 (1.0008, 1.003)	OR 1.002 *P* < 0.001 (1.001, 1.003)	OR 1.0008 *P* = 0.02 (1.0001, 1.001)	OR 1.001 *P* = 0.001 (1.0004, 1.001)

CR: cardiac rehabilitation; OR: odds ratio; CI: confidence interval.

Analyses adjusted for age, gender, comorbidity, CR duration, ethnicity, relationship status, employment, history of previous cardiac event, treatment received, baseline anxiety and depression score and year of initiating event.

Data were clustered with CR centres using cluster-robust standard errors.

**Table 4.** Results from multinomial logistic regression: CR wait time (late CR or CR wait time in days) and change in anxiety and depression category.

	Observed data				Imputed data			
Change in HADS category	Change in anxiety category RRR (95% CI)		Change in depression category RRR (95% CI)		Change in anxiety category RRR (95% CI)		Change in depression category RRR (95% CI)	
	Late CR	CR wait time	Late CR	CR wait time	Late CR	CR wait time	Late CR	CR wait time
Non-symptomatic to symptomatic	Reference group							
Symptomatic to non-symptomatic	0.85 *P* = 0.04 (0.74, 0.99)	0.99 *P* = 0.006 (0.994, 0.9991)	0.81 *P* = 0.01 (0.68, 0.95)	0.99 *P* = 0.46 (0.997, 1.001)	0.95 *P* = 0.37 (0.86, 1.05)	0.99 *P* = 0.16 (0.99, 1.00)	0.93 *P* = 0.20 (0.83, 1.03)	0.99 *P* = 0.85 (0.99, 1.00)
No change: symptomatic	1.04 *P* = 0.58 (0.89, 1.21)	0.99 *P* = 0.85 (0.997, 1.001)	1.06 *P* = 0.42 (0.90, 1.24)	1.002 *P* = 0.004 (1.0008, 1.004)	1.02 *P* = 0.58 (0.93, 1.13)	0.99 *P* = 0.96 (0.99, 1.00)	1.03 *P* = 0.59 (0.90, 1.18)	1.001 *P* = 0.16 (0.99, 1.00)
No change: non- symptomatic	0.93 *P* = 0.26 (0.82, 1.05)	0.99 *P* = 0.03 (0.996, 0.9998)	0.85 *P* = 0.01 (0.74, 0.97)	0.99 *P* = 0.09 (0.997, 1.0002)	0.98 *P* = 0.78 (0.90, 1.07)	0.99 *P* = 0.29 (0.99, 1.00)	0.94 *P* = 0.27 (0.85, 1.04)	0.99 *P* = 0.43 (0.99, 1.00)

RRR: relative risk ratio; CI: confidence interval; CR: cardiac rehabilitation.

Analyses adjusted for age, gender, comorbidity, CR duration, ethnicity, relationship status, employment, history of previous cardiac event, treatment received and year of initiating event.

Data were clustered with CR centres using cluster-robust standard errors.

For change in the anxiety category the findings were to the effect that delayed or increasing CR wait time is detrimental to mental health change from pre to post-CR. Statistically significant associations were observed for those who changed from the symptomatic to non-symptomatic category and those who remained non-symptomatic and CR wait time. For change in depression from pre to post-CR statistically significant associations were observed for those who changed from symptomatic to non-symptomatic, those who remained symptomatic and those who remained non-symptomatic and CR wait time. Testing model fit, 14 of the 16 Pearson chi-squared goodness-of-fit tests were non-significant (*P* > 0.39) indicating acceptable fit, but our model insufficiently predicted patients remaining depressed (*P* = 0.002 and *P* = 0.03 for continuous and dichotomised wait time models).

For the four anxiety and depression change categories: symptomatic to non-symptomatic, no change in symptomatic patients, non-symptomatic to symptomatic and remains non-symptomatic small changes in the predicted probabilities over time were found. For anxiety 12–10%, 14–17%, 5–7% and 67–64% at a wait time of 28 days and 168 days (24 weeks from referral), respectively. For depression 9% (no change over time), 6–10%, 4% (no change over time) and 79–75% at a wait time of 28 days and 168 days (24 weeks from referral), respectively (see Supplementary material).

Tables 3 and 4 also present the results based on the imputed data. These sensitivity analyses show that statistically significant associations between HADS category (post-CR) and CR wait time were observed, i.e. increasing CR wait time increases the likelihood of symptomatic HADS anxiety or depression scores (≥8) post-CR. For change in HADS category the findings were to the effect that delayed or increasing CR wait time is detrimental to mental health change from pre to post-CR; however, none of these effects reached statistical significance.

## Discussion

Current CR guidelines recommend the early commencement of CR when appropriate.^13,26,29,32,33^ However, evidence shows large inconsistencies across health regions and between patient groups, with variation in wait times which can exceed the required 4-week time frame.^31^ Inconsistencies in practice are concerning if there are implications to patient outcome. In this study, we explored participation in CR in those with and without symptoms of anxiety and depression and the relationship between CR wait time and HADS category (post HADS category and change in HADS category) after CR. The results of this study showed participation in CR varied by mental health status, in particular significantly lower completion rates were observed in those who were symptomatic. The likelihood of being classified as non-anxious or non-depressed post-CR was also improved when the commencement of CR was not delayed or had a reduced wait time. The results from the observed data, and in part from imputed data, support the requirement for timely commencement of CR. Furthermore, the sizable local practice variation, evident through high ICC values for wait time and programme duration, highlights that practices are not uniform across centres, and that further investigation of between-centre differences could play an important role to shed light on such delays or uncover new best practice examples.

When comparing the participation in CR services by HADS category at baseline and by change in HADS category the variation in median wait time was limited. However, wait time was still in excess of guidelines, which recommend CR commencement within 4 weeks of referral. As the data from these analyses has shown and in a previous analysis of CR wait time and physical activity outcomes,^24^ delays in starting CR can be detrimental to patient outcome so it is important to avoid delays which are not driven by clinical necessity. However, trials-based meta-analytical evidence has suggested that later psychological treatment initiation (>2 months post event) is more beneficial to mortality outcomes than early initiation.^38^ This shows that further research on the relationship between time to start of CR (psychological treatment initiation specifically) and a whole breadth of CR outcomes is needed.

In terms of the duration of CR some variation was observed in those who were symptomatic at baseline (1 day extra if anxious, 4 days extra if depressed) and by change in HADS category, e.g. those who remained non-symptomatic undertook shorter CR programmes than those who remained or developed mental health symptoms. It is unknown whether this substantially impacted patient care and outcome; however, the median programme duration for the population with HADS data (59 days) was below the recommended duration of CR, i.e. 12 weeks (84 days)^29,30,33^ and below the 2016 national UK average of 63 days.^31^ Although CR duration was longer in those who were symptomatic, the proportion of non-completers was also higher in those who had symptoms of anxiety (5% higher drop out) and in those with symptoms of depression (8% higher drop out). This seems to fit with previous research, which has reported drop out from CR is greater in those with higher anxiety and depression scores.^18^

With regard to associations between CR wait time and HADS outcome a relationship was observed in these analyses to the effect that the likelihood of having symptoms of anxiety or depression post-CR (HADS score ≥8) increases with every extra day between referral and start of CR. Similar effects were also observed when investigating CR wait time in accordance with guidelines defining ‘early CR’ (defined as 0–28 days) with a 13–24% increase in the likelihood of observing anxiety and depression symptoms following ‘delayed’ CR. The significant associations between CR wait time and HADS outcome remained, albeit the effects were smaller, when using imputed data, except when using timing as a categorical variable for anxiety, which did not reach significance. The impact of timing on outcome was also reflected in the predicted probability of being non-symptomatic, which decreased over time. Analyses by change in HADS category found significant associations for those changing from symptomatic and non-symptomatic for anxiety with both CR timing variables and for depression with the categorical timing variable only. As with the first analysis increasing or delayed CR wait time appears to impact change in outcome negatively. The results from the observed data indicate that programmes which fall outside wait time recommendations may inadvertently impact outcome with respect to HADS. However, analyses of change in HADS category using imputed data found only a negative trend for those changing from symptomatic and non-symptomatic for anxiety and depression with increasing or delayed CR wait time, and the results did not reach significance.

Although overall anxiety and depression scores were shown to reduce from pre to post-CR, not all programmes enter post-CR assessments onto NACR. In this study of those who had a baseline HADS score and had completed CR, 30% did not have a post-CR HADS score entered. A total of 21% of the population remain anxious or develop symptoms of anxiety post-CR (12% for depression), and this is associated with a heightened risk of mortality and re-occurrence of cardiovascular disease.^19–22^ Varying treatment approaches, i.e. dose and duration, could be explored to determine their impact on this subpopulation.

This study also highlights the need for improved clinical data capture, one aim of the British Association for Cardiovascular Prevention and Rehabilitation (BACPR)/NACR certification programme.^39^ Pre and post assessments using measures such as the HADS can be seen, by some, as posing a substantial time burden on patients and services; however, a tailored intervention with guided long-term management is the cornerstone of effective CR.^13,33^ Newer technologies using computerised adaptive testing systems that have been used successfully in similarly challenging areas such as cancer/palliative care^40,41^ are also under development for CR,^42,43^ and provide future ways to less burdensome but accurate approaches to assess patients’ mental health. Incentive-based approaches to improve data capture could also be considered, but may not be the most powerful motivator as noted in a recent report by the Healthcare Quality Improvement Partnership (HQIP) on engaging clinicians in quality improvement through audit.^44^

### Limitations

This UK-based analysis represents a large and current investigation into the impact of CR wait time on anxiety and depression outcomes in routine practice, a clear strength of this study. Known relevant confounding variables and data clustering were managed effectively, although it is acknowledged that a measure of disease severity was not included in this analysis as this is not collected in NACR. The main limitation of this analysis is the lack of consistent assessment and documentation of mental health outcomes even for audit purposes. Some of the missing data is due to participants not completing their CR programme, thereby missing follow-up assessment, while some is due to services collecting outcomes with other measures (including the PHQ-9 would have increased the sample size by 983 and by 978 for GAD7 but the majority of the loss is due to services’ documentation practices. As outlined in the introduction, mental health outcomes merit attention, because they are predictive of mid and long-term cardiac events including evidence that depression and anxiety are differentially predictive of these.^8,9^ For many patients post-CR data were not available, which is troubling because our results show that a sizable share of patients potentially deteriorate in their mental health status (Table 2). Overall, this points to the importance of ensuring high data quality in audits for all clinically important variables. Finally, the results of these analyses have only been determined with one specific instrument, the HADS. In 2000 the National Framework for Coronary Heart Disease was published by the Department of Health, setting standards for modern practice including the use of HADS.^45^ Since then HADS has been the preferred clinical screening tool. Nevertheless, evidence is increasingly questioning whether the HADS is the most optimal choice for screening;^46,47^ therefore, results need to be replicated with other instruments.

## Conclusions

Audit of CR services shows variation in service delivery and in some cases practice, which falls outside of recommended guidelines. In these analyses, CR wait time has been shown to predict the outcome of anxiety and depression status to the extent that delays in starting CR are detrimental. Programmes falling outside the 4-week window for commencement of CR following referral must strive to reduce wait times to avoid negative impacts to patient outcome.

## Supplementary Material

Supplementary material

Supplementary material
