# Supplementary figures and images for: Does service timing matter for psychological outcomes in cardiac rehabilitation? Insights from the National Audit of Cardiac Rehabilitation
Source: Eur J Prev Cardiol. 2017 Nov 9;25(1):19–28. doi: 10.1177/2047487317740951 (PMC5757407; doi:10.1177/2047487317740951)

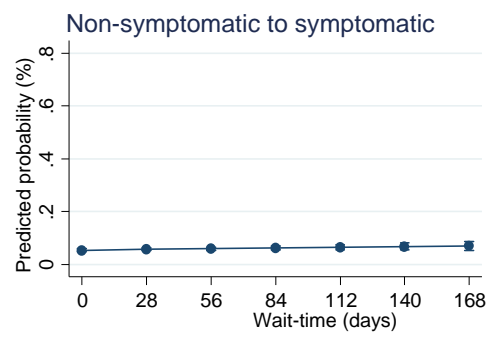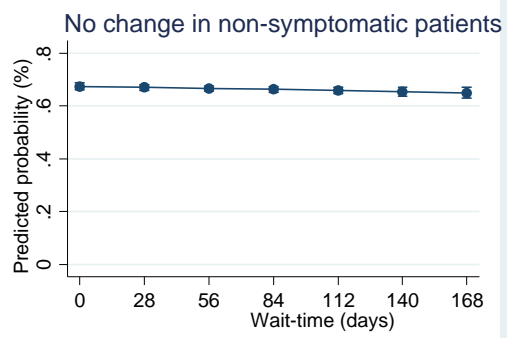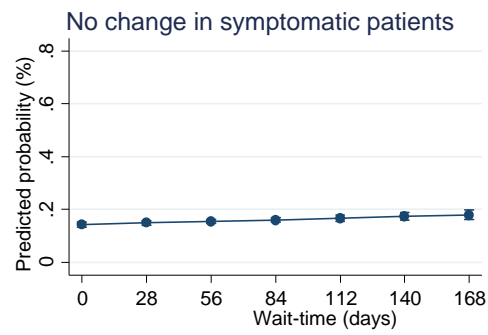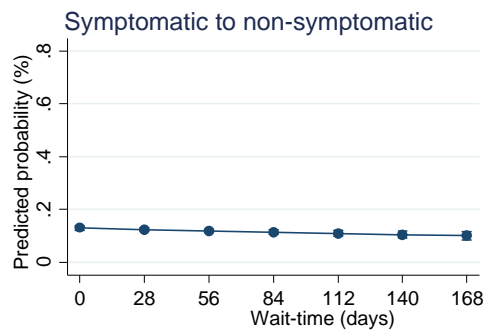

Supplement: Supplementary material [file Supplement_Anxiety.pdf]

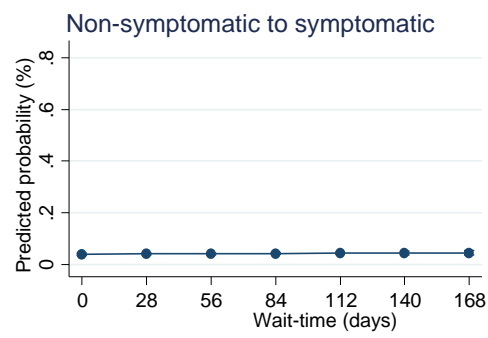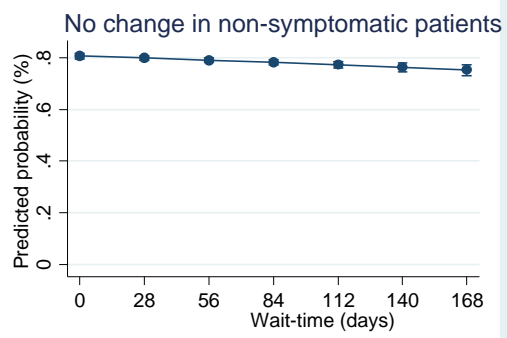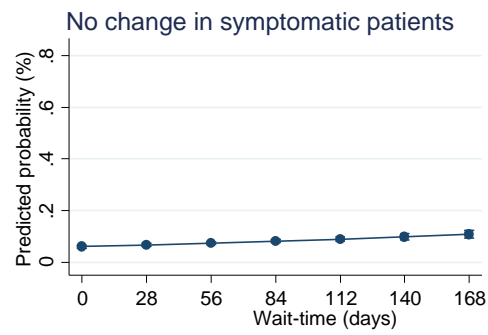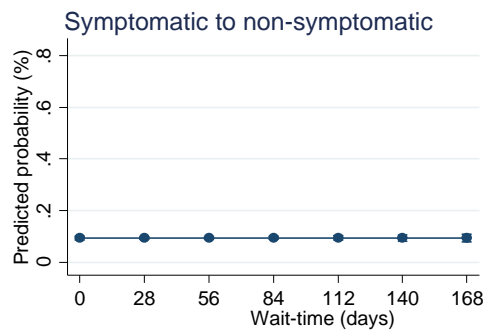

Supplement: Supplementary material [file Supplement_Depression.pdf]
